# Prader–Willi-Like Phenotype Caused by an Atypical 15q11.2 Microdeletion

**DOI:** 10.3390/genes11020128

**Published:** 2020-01-25

**Authors:** Qiming Tan, Kathryn J. Potter, Lisa Cole Burnett, Camila E. Orsso, Mark Inman, Davis C. Ryman, Andrea M. Haqq

**Affiliations:** 1 Department of Pediatrics, University of Alberta, Edmonton, AB T6G 2E1, Canada; qtan3@ualberta.ca; 2 University of Alberta Hospital, Stollery Children’s Hospital, Edmonton, AB T6G 2B7, Canada; Kathryn.Potter@albertahealthservices.ca; 3 Levo Therapeutics, Inc., Skokie, IL 60077, USA; lcburnett@levotx.com (L.C.B.); dryman@levotx.com (D.C.R.); 4 Department of Agricultural, Food and Nutritional Science, University of Alberta, Edmonton, AB T6G 2E1, Canada; orsso@ualberta.ca; 5 Department of Pediatrics, University of Saskatchewan, Saskatoon, SK S7N 0W8, Canada; mark.inman@usask.ca

**Keywords:** Prader–Willi, 15q11.2, *SNORD116*, atypical microdeletion

## Abstract

We report a 17-year-old boy who met most of the major Prader–Willi syndrome (PWS) diagnostic criteria, including infantile hypotonia and poor feeding followed by hyperphagia, early-onset morbid obesity, delayed development, and characteristic facial features. However, unlike many children with PWS, he had spontaneous onset of puberty and reached a tall adult stature without growth hormone replacement therapy. A phenotype-driven genetic analysis using exome sequencing identified a heterozygous microdeletion of 71 kb in size at chr15:25,296,613-25,367,633, genome build hg 19. This deletion does not affect the *SNURF-SNRPN* locus, but results in the loss of several of the PWS-associated non-coding RNA species, including the *SNORD116* cluster. We compared with six previous reports of patients with PWS who carried small atypical deletions encompassing the snoRNA *SNORD116* cluster. These patients share similar core symptoms of PWS while displaying some atypical features, suggesting that other genes in the region may make lesser phenotypic contributions. Altogether, these rare cases provide convincing evidence that loss of the paternal copy of the *SNORD116* snoRNA is sufficient to cause most of the major clinical features of PWS.

## 1. Introduction

Prader–Willi syndrome (PWS) is an imprinted disorder affecting many organ systems, with a frequency of about 1 in 10,000 to 20,000 live births [1]. Major characteristics of PWS include infantile lethargy and hypotonia causing poor feeding and failure to thrive, followed by excess weight gain and onset of hyperphagia in early childhood, in addition to hypogonadism, short stature, delayed development, minor facial abnormalities, cognitive impairment, and behavioral and psychiatric disturbances [2]. PWS is caused by an absence of a functionally active paternal contribution in the chromosome 15q11.2-q13 region [2] via three distinct genetic mechanisms: large deletions (65–75%), maternal uniparental disomy (UPD; 20–30%), and imprinting defects (ID; 1–3%) [3]. More than 99% of cases can be easily detected by DNA methylation analysis of abnormal parent-specific imprinting within the Prader–Willi critical region on chromosome 15 [4]. The deletion class is subdivided into the typical Type 1 or Type 2 deletion, both of which are almost always de novo events [3]. The larger Type 1 deletion involves breakpoints (BP) BP1 and the distally located BP3 (~6 Mb), whereas Type 2 covers BP2 to BP3 (~5.6 Mb). In rare cases, both larger and smaller deletions in PWS than typically described have been identified, which may show distinct phenotypic features [3]. 

**Figure 1:**
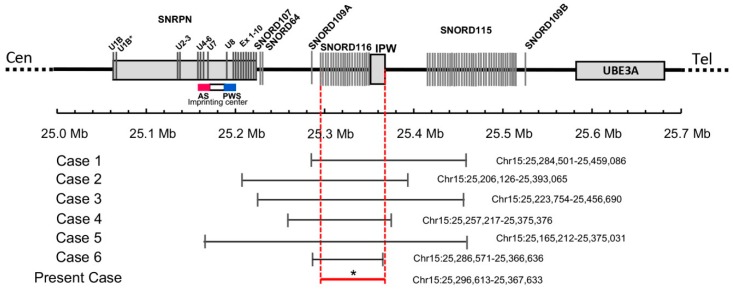
Schematic map of the 15q11.2 region between the *SNRPN* and *UBE3A* genes, which is represented at scale with physical distance in Mb. The reported deletions are shown at the bottom drawn to scale and depicting genomic coordinates for build hg19. * The exact breakpoints of the deletion in the present case are not available. The minimal critical region spans from 25296613bp to 25367633pb as shown by the red horizontal line.

Here, we report a 17-year-old boy who met six of the seven major revised clinical criteria for diagnosis of Prader–Willi syndrome, including neonatal hypotonia, feeding difficulties and failure to thrive as an infant, excess weight gain and hyperphagia by age 3, global developmental delay, and dysmorphic facial features. Unlike many children with PWS, from age 4 onward, his height followed the 90–95th percentiles, and he had spontaneous onset of puberty by age 15. Methylation analysis of the *SNURF-SNRPN* exon 1 region showed a normal pattern in the proband. However, further genetic analysis using exome sequencing identified a heterozygous microdeletion of 71 kb in size, spanning at least chr15:25296613-25367633 (genome build hg 19; Figure 1). This deletion results in the loss of several of the PWS-associated non-coding RNA species, including the *SNORD116* cluster. In the literature, only six PWS cases that were caused by small atypical deletions spanning snoRNAs at 15q11.2 have been reported before, with deletion sizes ranging from 80 to 236 kb (see Table 1), all of which overlap the *SNORD116* region [5,6,7,8,9,10]. Altogether, these rare cases provide further insights into genotype–phenotype correlations.

## 2. Materials and Methods 

Given that the patient’s rare phenotype had remained unexplained, clinical-grade exome sequencing with deletion and duplication analysis was done (Fulgent Genetics, Temple City, California, CA, USA) [11]. Rare variant and del/dup analysis of 4637 genes was guided by the phenotype, focusing on genes annotated to intellectual delay, hypotonia, obesity, and other features matching the patient’s clinical presentation. Genomic DNA was isolated from whole blood using an automated DNA extraction machine (AnaPrep system, BioChain, Newark, DE, United States). DNA was sheared and barcoded using the Illumina TrusightOne Kit, and enriched for the coding exons of targeted genes using hybrid capture technology. Prepared DNA libraries were then sequenced using the Next Generation Sequencing technology.

Following alignment, variants were called in regions of at least 10× coverage. For this specimen, 99.3% and 98.3% of the coding regions and splicing junctions of the genes listed had achieved coverage of at least 10× and 20×, respectively. Regions that did not reach 10× coverage were not evaluated, unless they could be filled in by targeted Sanger sequencing assays. Variants were annotated using locus-specific databases, literature searches, and manual curation. The *SNORD116* cluster deletion was identified by Fulgent Genetics’ proprietary copy number detection algorithm and pipeline; precise breakpoints could not be established using this pipeline. Only variants classified as pathogenic, likely-pathogenic, or of unknown significance, which were thought to be related to the patient’s phenotype, were reported [12]. Any reported variants that had quality scores less than 500 (roughly 40× of the coverage for a heterozygous variant) were confirmed by Sanger sequencing. All genes listed were evaluated for large deletions and/or duplications in regions of the genes with significant pseudogenes. 

Additionally, real-time quantitative polymerase chain reaction (qPCR) analyses were done to quantify *SNORD116* allele number in the patient and his parents. DNA was amplified for the targeted region of the *SNORD116* microdeletion and quantified using a QuantStudio 6 instrument. Detection of PCR products was enabled by the inclusion of a fluorescent reporter molecule in each reaction well that yielded increased fluorescence in real time as product DNA accumulated. Signal strength for the reactions targeting *SNORD116* were compared to control genes and control individuals, to assess the presence of the haploid versus diploid copy number.

## 3. Results

### 3.1. Clinical History

A Caucasian boy was born at 38 weeks’ gestation through cesarean section, with appropriate weight (3140 g; 18th percentile, Z = −0.9) and length (51 cm; 50th percentile, Z = 0.0) for gestational age [13]. There were no complications during pregnancy. The patient received resuscitation after birth due to marked hypotonia and low respiratory effort. He stayed in the neonatal intensive care unit for three weeks and required nasogastric (NG) tube feeding because of prominent hypotonia, extreme lethargy, and severe failure to thrive as an infant. A gastrostomy tube (G-tube) was placed as a result of aspiration pneumonia at 6 months of age, and reversed at age 3. He had global development delay and walked at 20 months. He started to say “mama" and "dada” at 18 months and speak in sentences at 40 months of age. In Grade 7, difficulties in learning, especially in reading and mathematics, were present. Due to poor academic performance in school and his history of medical concerns, he was referred for a psycho-educational assessment. The Wechsler Intelligence Scale for Children® Fifth Edition test results indicated that he had processing speed deficits (Processing Speed Index = 6th percentile), interfering with the encoding, process and retrieval of information; however, his overall cognitive ability was in the average range (53rd percentile). Nevertheless, he was mainstreamed in normal education programs. He was noted to have central sleep apnea since birth and has been on bilevel positive airway pressure therapy (BiPAP) since the age of 6. He had orchidopexy surgeries when he was 22 and 30 months of age and had a tonsillectomy and adenoidectomy at age 3. 

Hyperphagia and excess weight gain were first noted at age 3 years. The boy experienced an unrelenting feeling of hunger but did not often engage in food-seeking behaviors. From age 4 onward, his body weight and body mass index (BMI) remained above the 97th percentile for age. Strict control of food access and energy-restricted diets (1000–1200 calories per day) had stabilized his BMI at 28.45 kg/m^2^ (97.8%, Z = 2.01; Figure S1). Meanwhile, his height followed the 90–95th percentiles, and he reached a tall adult stature (181.4 cm, 81.0%, Z = 0.88; mid-parental height is 181.5 cm) without the use of growth hormone (GH) replacement therapy, which is atypical for PWS. His hands (19 cm) and feet (27 cm) are of normal size. He exhibited characteristic facial features, including a narrow bitemporal diameter of the skull, almond shaped eyes, and a thin and down-turned upper lip. The boy had ongoing behavioral problems including rigidity and intolerance of changes in routine. He also presented with frequent skin picking to the point of bleeding. Levothyroxine was given from age 14 years 11 months to 17 years for his fatigue and possible central hypothyroidism (FreeT4 = 10.1 pmol/L). Fluoxetine was used to treat his depression, anxiety, obsessive compulsive disorder (OCD), and suicidal thoughts. He was prescribed atomoxetine for attention deficit hyperactivity disorder (ADHD), depression, social anxiety disorder, and autism spectrum disorder (Level 1). Lamotrigine was given as a mood stabilizing adjunct for his major depression. In addition, he was in regular consultation with a psychiatrist for behavioral and emotional management. 

The patient was referred to our clinic at age 15, by which his puberty was complete. Unlike many children with PWS, he had spontaneous onset of puberty. He had Tanner 5 pubic hair and his testes were 20 mL bilaterally. Previous urology notes indicated that he was in puberty at age 11.5. His left testis was >3–4 mL (~Tanner 2). At age 12 years 9 months, he had Tanner 4 pubic hair; his left testis was 10 mL and the right one was not-palpable. Laboratory results showed normal thyroid function on levothyroxine (low-normal Free T4 = 10.1–12.7 pmol/L), as well as normal hemoglobin A1C, morning cortisol, and fasting lipid profile; testosterone level was consistent with puberty. There was no history or symptoms of diabetes or adrenal insufficiency. He had no issues with headaches or vision problems. His parents were healthy; they previously had a baby girl who was stillborn at 38 weeks. The patient’s older brother and younger sister were healthy with no known genetic diagnosis. His maternal and paternal grandfathers were obese, but there is no known family history of morbid obesity, genetic syndromes, hypotonia, or mental retardation. 

### 3.2. Genetic Findings

Because of his neonatal hypotonia, the boy had diagnostic testing targeting the PWS critical region. However, DNA methylation analysis of the PWS-imprinting center showed a normal methylation pattern, and chromosome analysis showed a normal male karyotype. Testing for spinal muscular atrophy (SMA) and myotonic dystrophy type 1 were also negative. In addition, his muscle biopsy and brain magnetic resonance imaging (MRI) were normal. He was re-referred for genetic counseling at age 6 years because his phenotype was more suggestive of PWS than of any other recognizable disorder, and testing for the fragile X expansion was negative. The patient was assessed as having the phenotype of PWS without a genetic confirmation. Neuromuscular workup between ages 12 and 13, done because of ongoing hypotonia, was normal. Following that, clinical-grade exome sequencing was performed, and this identified the *SNORD116* microdeletion.

A heterozygous deletion distal to the *SNRPN* gene in the PWS critical region at 15q11.2 was identified in the submitted specimen. The exact breakpoints of this deletion were not discernible from the present analysis. The deletion spans the following region in the GRCh37/hg19 build of the human genome: chr15: 25296613-25367633. This deletion does not affect the *SNURF-SNRPN* locus; however, it leads to the loss of a number of non-coding RNA species which have been implicated in PWS, or a PWS-like phenotype, including the *SNORD116* cluster (PubMed: 19498035, 20588305, 18500341). Subsequent parental testing showed that the parents did not carry this microdeletion, suggesting that the microdeletion found in the patient was de novo and had occurred on the paternally transmitted allele.

## 4. Discussion

We describe here a rare atypical case, in which a 71 kb microdeletion at 15q11.2 involving the *SNORD116* complex results in a Prader–Willi-like phenotype. The patient exhibited many of the major clinical diagnostic criteria for PWS [14], including neonatal and infantile hypotonia, feeding difficulties that required tube feeding for the first 3 years of life, followed by the onset of excess weight gain and hyperphagia, global developmental delay, and characteristic facial features. Additional features included sleep apnea, behavioral problems, skin picking, and speech delay. Hypogonadism is a common clinical feature of PWS but not seen in this patient. Individuals with PWS commonly fail to spontaneously complete puberty. They have a typical pattern of growth, which is characterized by decreased height velocity in childhood, absence of a pubertal growth spurt, and compromised final adult height [15]. Interestingly, this patient had spontaneous progression through puberty and reached a tall adult stature without GH treatment. 

The minimal critical region for PWS is proposed to be approximately 95 kb in size (at chr15:25280000-25375000, genome build hg 19) containing two C/D box snoRNAs: the *SNORD116* cluster and *SNORD109A*, as the only putative functional genes [9]. In review of the regions of deletion in the present and previously described cases that cause the key characteristics of the PWS phenotype, the *SNORD116* cluster, *SNORD109A* and the Imprinted in Prader–Willi (*IPW*) exons were found to be consistently deleted. Additionally, in four of the seven subjects, at least one of the adjacent genes, including the *SNURF-SNRPN* locus, *SNORD107*, *SNORD64*, *SNORD108*, and *SNORD115* cluster, was also deleted [5,6,7,9]. The microdeletion in our patient is very similar to the cases reported by Bieth et al. [8] and Fontana et al. [10]. The deletion in Bieth’s subject was 118 kb in size, encompassing the *SNORD109A* gene, the whole *SNORD116* cluster, and the non-coding exons of the *IPW* transcript. The subject in Fontana’s report had an 80 kb microdeletion that was restricted to the *SNORD109A* gene, the complete *SNORD116* cluster, and the first 2 exons and part of exon 3 of the *IPW* gene. The two subjects and our patient shared a core phenotype, which is characterized by neonatal hypotonia, excess weight gain, hyperphagia, global development delay, and distinctive facial features, pointing towards a causative role for the genes in the minimal critical region in the broader phenotype of typical PWS. Further, in animal models of PWS, knockout *Snord116* mice displayed cognitive deficits [16], growth retardation [17], hyperphagia, and marked obesity [18]. However, the role of *SNORD109A* in the clinical manifestations of PWS has not been examined as no mouse orthologs have been identified for this gene [19]. Nevertheless, the current findings demonstrate that the deficiency of *SNORD116* snoRNA is sufficient to result in hyperphagia, obesity, developmental delay, and other clinical manifestations associated with PWS, while additional genes in the region may contribute.

The microdeletion in our patient is the shortest among those in the six previously reported subjects; these patients, however, showed no obvious clinical differences. Some features appear to be less constant in these individuals compared to those with typical PWS deletions. Patients with PWS commonly fail to spontaneously complete puberty. Impaired linear growth is frequently observed in PWS with approximately 90% of affected individuals being short in stature without GH treatment. Like those with the typical genetic lesions seen in PWS, the subjects reported by de Smith et al. [6], Bieth et al. [8], and Hassan et al. [9] had delayed or incomplete puberty and short adult heights (below the 25th percentile). Interestingly, our patient had spontaneous puberty and reached an adult height above the 95th percentile. The subject with PWS reported by Fontana et al. [10] had delayed puberty but his adult stature was within the normal range (174 cm; between the 25th and 50th percentile). He also had normal sized hands and feet and intellectual development (intelligence quotient (IQ) of 80) with mild learning disability, which is very similar to our patient. There were no feeding problems during infancy. He had no sleep disturbances, skin picking or other behavioral problems. Of note, in our patient and the ones reported by Bieth et al. [8] and Fontana et al. [10], food-related behavioral problems (e.g., foraging and sneaking) were milder than typically seen in individuals with PWS. They had the lowest BMIs at 31, 28.45, and 25.1, respectively (through dietary restriction), with a range of 39–50 for the other subjects. These atypical findings indicate that certain genes in the PWS critical region may make more subtle contributions to traits, resulting in a milder phenotype.

In summary, we report on a patient with a Prader–Willi-like phenotype caused by an atypical microdeletion in the PWS critical region. Our findings provide further evidence that deletion of the *SNORD116* region is sufficient to cause the key characteristics of PWS, although some atypical features, including tall stature and spontaneous complete puberty, suggest that other genes in the region may make lesser phenotypic contributions. More research is needed to better understand the role of *SNORD116* in human energy homeostasis and growth. 

## Figures and Tables

**Table 1:** Comparison of present case with previously published cases with *SNORD116* microdeletion.

	Case 1 [5]	Case 2 [6]	Case 3 [7]	Case 4 [8]	Case 5 [9]	Case 6 [10]	Present case
Deletion size (kbp)	175	187	236	118	210	80	71
Ethnicity	Caucasian	South Asian Indian	African-American	Caucasian	Caucasian	Caucasian	Caucasian
Gender	Male	Male	Male	Female	Female	Male	Male
Birth weight (g)	3218	2800	3020	2780	3334	2710	3140
Birth length (cm)	54.5	N/A	53	48	54.6	49	51
Age at examination (years)	4.8	19.5	11	23	26	18	17
**Clinical features**							
Hypotonia	+	+	+	+	+	+	+
Infantile feeding problems/FTT	+	+	+	+	+	−	+
Tube feeding	+	−	+	+	−	−	+
Start of excess weight gain (months)	18	24	6	18	30	Between 48–72	36
Hyperphagia	+	+	+	+	+	+	+
Overweight/Obesity	+	+	+	+	+	+	+
Distinctive facial features	+	N/A	+	+	+	+	+
Hypogonadism	+	+	+	+	N/A	+	−
Developmental delay	+	+	+	+	+	+	+
Mental retardation	+	+	+	+	N/A	−	−
Behavioral problems	+	+	+	+	+	−	+
Skin picking	+	+	−	+	+	−	+
Sleep disturbances/ apnea	+	N/A	+	+	N/A	−	+
Short stature	−	+	+	+	+	−	−
Small hands/feet for height	+	+	−	N/A	+	−	−
Eye abnormalities	−	N/A	+	N/A	N/A	+	−

FTT: failure to thrive; N/A: not available; +: present PWS characteristic; −: absent PWS characteristic.

## Supplementary Materials

The following are available online at https://www.mdpi.com/2073-4425/11/2/128/s1, Figure S1: (a) BMI curve and (b) height and weight evolution plotted on World Health Organization Growth Charts for Canada.

## Diagnostic Testing

DNA-level diagnostics were performed by Fulgent Genetics (Temple City, California).
