# Supplementary material for: Prader–Willi-Like Phenotype Caused by an Atypical 15q11.2 Microdeletion
Source: Genes (Basel). 2020 Jan 25;11(2):128. doi: 10.3390/genes11020128 (PMC7073628; doi:10.3390/genes11020128)

genes

MDPI

SUPPLEMENTARY FIGURES

Figure S1: (a) BMI curve and (b) height and weight evolution plotted on World Health Organization Growth Charts for Canada.

a. **b.**

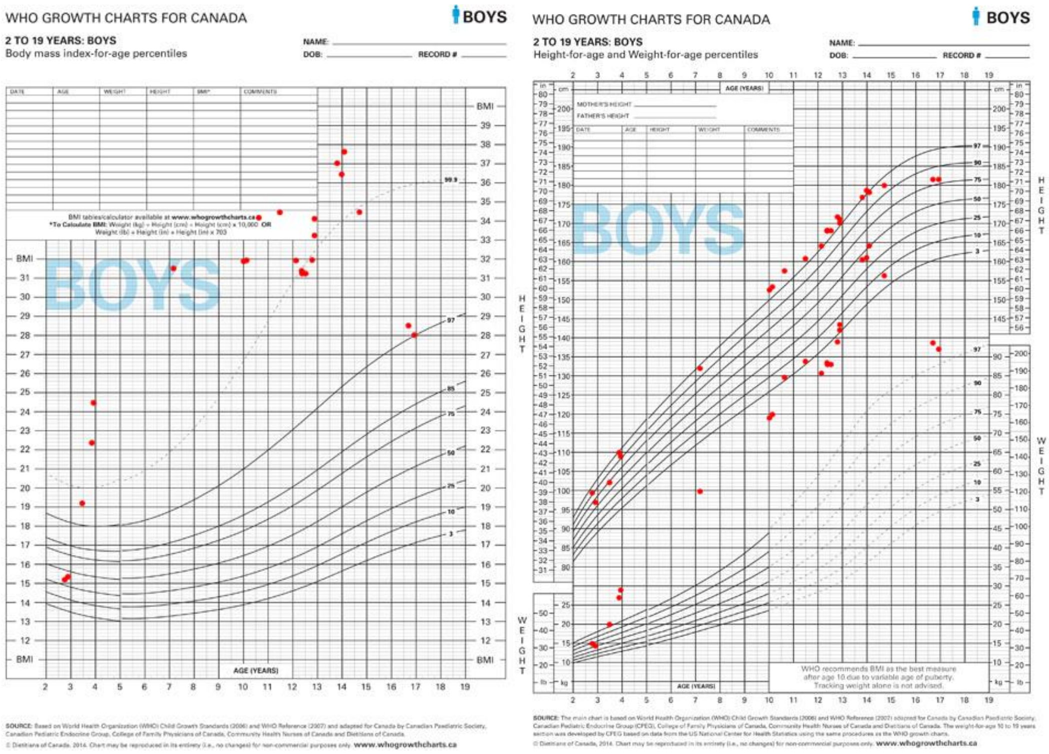

Supplement: Supplementary file 1 [file genes-11-00128-s001.pdf]
